# ‘Don’t forget the children’: a qualitative study when a parent is at end of life from cancer

**DOI:** 10.1007/s00520-021-06341-3

**Published:** 2021-06-18

**Authors:** Eilís McCaughan, Cherith J. Semple, Jeffrey R. Hanna

**Affiliations:** 1 School of Nursing, Ulster University, Cromore Road, Coleraine, BT52 1SA Co. L’Derry UK; 2 School of Nursing, Ulster University, Shore Road, Newtownabbey, BT37 0QB Co, Antrim UK; 3 South Eastern Health and Social Care Trust, Cancer Services, Ulster Hospital, Upper Newtownards Road, Belfast, BT16 1RH UK; 4 Department of Psychiatry, Warneford Hospital, University of Oxford, Oxford, OX3 7JX UK

**Keywords:** End of life, Psychosocial support, Parental life-limiting illness, Parental cancer, Dependent children, Qualitative study

## Abstract

**Purpose:**

Preparation for end of life is one of the greatest challenges faced by parents with cancer who have dependent children (< 18 years old), with requirement for support from professionals. The aim of this study is to explore how parents can be best supported in relation to their children, when a parent is at end of life from cancer.

**Methods:**

This is an interpretive qualitative study, using 79 semi-structured interviews with parents at end of life (n3), bereaved parents (n21), health and social care professionals (HSCPs) (n32) and funeral directors (n23). Data were analysed thematically and triangulated.

**Results:**

Parents are central to preparing their children for the death of a parent. Striving for everyday ordinariness, maximising social networks, maintaining hope and making preparations for the future are helpful for families when a parent is at end of life. Most HSCPs were unaware of the challenges faced by parents at end of life, and psychosocial support was often left outside the caring realm. As a result, funeral directors noted complexities faced by the families after the death. Results are discussed under four themes: (1) communication with the children as a process, (2) coping throughout the unfolding end of life experience, (3) tension and complexities at end of life and (4) preparing for the future.

**Conclusions:**

Parents should be reassured that by involving the children early in the end of life experience when the ill-parent is ‘well enough’ to parent enables them to be actively involved in supporting their child through one of the greatest life changing event. A number of recommendations are discussed for professionals.

**Supplementary Information:**

The online version contains supplementary material available at 10.1007/s00520-021-06341-3.

## Background

Preparation for end of life (EOL) is one of the greatest challenges faced by parents with cancer who have dependent children (< 18 years old) [1, 2]. Parents often delay telling children that their parent’s cancer is incurable and they will die from this illness, perceiving this as protective for the children [3]. Advance planning is further hindered if parents do not wish to acknowledge the inevitability of death [4]. Children desire to be informed of their parent’s poor cancer prognosis [5] and want regular updates surrounding their parent’s treatment and declining health [6] and to be involved in EOL care [7, 8].

Studies demonstrate that children less prepared for the death of a parent are more susceptive to adverse psychological adjustment in the short term and later life [9, 10]. To promote children’s ability to manage and adapt to this difficult situation, Walsh’s family resilience theory [11] highlights that clear and honest communication couched within a cohesive family network is fundamentally important [2, 12–15].

To facilitate family resilience, there is often a need for parents to be encouraged, equipped and supported to meet the needs of their children throughout the EOL experience [16]. Providing support for children’s parents not only enhances a parent’s capacity to support their children through this highly stressful life-event, but can also be emotionally protective for them as caregivers [17, 18].

Although health and social care professionals (HSCPs) are often well placed to support families at EOL and through the immediate bereavement period, this is often fraught with complexities [19, 20]. The immediate bereavement period is from the time of death to the funeral that follows [19]. Some of the complexities include prognostication being inherently difficult and the management of patients often complex clinical condition at EOL presiding over the needs of the well-parent and children [21]. Studies highlight insufficient training for HSCPs; therefore, they lack skills, competence and confidence in addressing parents and children’s need for support around the time of parental death from cancer [20], with some fearing they could make the situation worse [22]. This is despite national and international EOL guidelines repeatedly acknowledging that families should have honest, sensitive and well-informed conversations about dying, death and bereavement [23].

### Aims and objectives

This study aims to explore how parents can be best supported in relation to their dependent children, when a parent is at EOL from cancer. The objectives of this study are to investigate the following:

o The experience of parents as they prepare/prepared for the death of a parent with cancer who has dependent children
p Parents’ perception of need as they prepare and support their dependent children for the death of a parent from cancer
q Professionals’ experiences and perceptions of supporting parents when a parent of dependent children is at EOL from cancer and through the immediate bereavement period

## Methods

An interpretative qualitative study design was adopted [24]. This design provided flexibility for the researchers to follow-up on identified categories between and within the study populations throughout the data collection process [25]. To validate the findings from the sample, data were triangulated to enhance the credibility of the study [26].

### Participants

Seventy-nine participants were involved in this study, comprising of parents at EOL (n3), bereaved parents with dependent children (n21), HSCPs from one healthcare Trust (n32) and funeral directors from urban and rural settings (n23). Using convenience and purposive sampling, participants were recruited by the second and third authors. Volunteer sampling techniques were used to aid recruitment of parents (EOL and bereaved) to the study. An outline of the inclusion and exclusion criteria and participant characteristics of the sample are provided in Table 1.

**Table 1:** Outline characteristics of the 79 participants recruited in the study

Variables	Parents at end of life (n3)	Bereaved parents (n21)	HSCPs (n32)	Funeral directors (n23)
Inclusion criteria	- Awareness of their poor prognosis - Considered physically well to participate* - Dependent children (< 18 years old) - Resided in Northern Ireland**	- Experienced the death of a co-parent to cancer**** - Dependent children (< 18 years old) at the time of death - Resided in Northern Ireland**	- HSCPs who provide care to end-stage cancer patients as part of their clinical practice	- Funeral directors from private and public limited companies between rural and urban locations in Northern Ireland
Exclusion criteria	- Parents with gross psychopathology***	- Parents with gross psychopathology***	- HSCPs who do not work within oncology departments or provide EOL care	- Funeral directors outside of Northern Ireland**
Participants	Mother (n = 0) Father (n = 3)	Mother (n = 12) Father (n = 9)	Acute specialists Palliative social worker (n = 2) Palliative clinical nurse specialist (n = 2) Palliative care consultant (n = 3) Acute clinical nurse specialist (n = 1) Oncology physiotherapist (n = 1) Oncology clinical nurse specialist (n = 3) Community specialists Community clinical nurse specialist (n = 1) Oncology physiotherapist (n = 1) Speech and language therapist (n = 1) Occupational therapist (n = 1) Palliative care educationalist (n = 2) Acute generalists Acute care nurse (n = 3) Chemotherapy nurse (n = 2) Community generalists Community care nurse (n = 9)	Male (n = 19) Female (n = 4)
Ethnicity	White (n = 3)	White (n = 20) Asian (n = 1)	White (n = 30) Asian (n = 2)	White (n = 23)
Gender/age of children	Boy, 0–11 years old (n = 0) Boy, 12–18 years old (n = 2) Girl, 0–11 years old (n = 0) Girl, 12–18 years old (n = 3)	Boy, 0–11 years old (n = 15) Boy, 12–18 years old (n = 7) Girl, 0–11 years old (n = 19) Girl, 12–18 years old (n = 12)	x	x
Recruitment	One hospice service (n = 1) One family support services (n = 1) Public advert (n = 1)	One hospice service (n = 3) Two family support services (n = 14) Public advert (n = 4)	One Trust in United Kingdom (n = 32)	Rural, private limited companies (n = 10) Urban, private limited companies (n = 5) Rural, public limited companies (n = 3) Urban, public limited companies (n = 5)

* Various side-effects from treatments or a health decline may have made it difficult or too demanding for parents to participate in the study.

** This was a Northern Irish-based study.

*** Ethical principle of non-maleficence.

**** To promote participant autonomy, no limits were applied regarding period between death and inclusion to the study.

### Data collection

Semi-structured interviews were conducted between February 2018 and February 2020. Data collection was guided by topic guides and informed by the literature alongside the research and expert group. The expert group consisted of a palliative care social worker and clinical nurse specialist, a family support worker and a bereaved parent and child (aged 14). The guides were iteratively modified as necessary between and within sample groups to enable follow-up with identified categories in subsequent interviews. Interviews lasted between 20 and 120 min and were conducted face-to-face by the authors who had no prior relationships with the participants. Interviews were completed when no further categories were identified.

### Data analysis

Audio-recordings were transcribed verbatim by the third author and verified by the research team. Braun and Clarke’s thematic analysis framework [27] was used to analyse the data generated from the study populations. One author read and reread the transcripts of each study population to gain a sense of each participant’s story. Following line-by-line scrutiny of the transcripts, the same author coded the data using NVivo V12 by marking similar phrases or words in the narratives. Subsequently, data from parents at EOL, bereaved parents, HSCPs and funeral directors were triangulated to provide a broader and enhanced understanding on how best parents can best supported as they prepare and support their children for the death of a parent from cancer [28]. Deployed as an inductive method, JRH identified similarities and differences in the data between the sample and where some of them merged into themes. To ensure rigour and trustworthiness, the data were independently analysed by EMcC and CJS. Themes were verified and refined through critical dialogue with all authors.

### Ethical considerations

Participants received oral and written information about the study and provided written consent. Participants were made aware of their right to withdraw, and assurances of confidentiality were given. To protect the anonymity of participants, personal identifiable information was removed by the JRH who transcribed the audio-recordings, and pseudonyms were used in the reporting of this study. A distress protocol was established, and a support pack was provided to participants as part of the debriefing process. Ethical approvals were obtained.

## Results

Overall, four themes were identified from the data: (1) communication with the children as a process, (2) coping throughout the unfolding EOL experience, (3) tension and complexities at EOL and (4) preparing for the future.

### Theme 1: communication with the children as a process

Parents (this term is referred to when the finding is representative of data from both parents at EOL and bereaved parents) reported their need for time to ‘digest’ the shock that the cancer was incurable, before they considered telling the children. HSCPs did not acknowledge this as a factor, but when probed as to why parents may delay sharing this news with the children, HSCPs perceived parents were in denial surrounding the reality of the situation. However, most parents reported an awareness that death was going to be the inevitable outcome.

Your head is in a spin and yes you are thinking about your kids but there was so much to process and take in before telling them. [Interview 20, bereaved mother].

Communication between the parents and children regarding mum or dad’s prognosis was generally an ongoing process throughout the EOL experience, with key conversations to be had at different time points. These included sharing the poor prognosis with the children, telling the children their mum or dad is going to die soon and preparing the children for the actual death. While some parents sought out advice from a family support service or the Internet, most parents ‘muddled’ through challenging conversations with the children alone. However, HSCPs did not routinely intervene or explore with the parent how they were going to tell the children, and the problem was more often left outside the caring realm.

I wish it was more joined up and thinking about us as a family unit. I understand their role was helping Philip and making sure his care was provided and he was looked after. But I was left to pick up the pieces. [Interview 12, bereaved mother].

The parent data evidenced that telling the children that mum or dad’s death was going to happen was perpetually at the forefront of parents’ mind. How to tell the children this news seemed a very challenging activity and to be avoided if possible. Some HSCPs (mainly those with clinical experience and personal exposure to death and dying) ‘were on the case’ and had actively steered the parents through a systematic approach of telling the children that mum or dad was eventually going to die from their cancer.

It’s one of the most difficult things a parent will go through, but I’ve seen the aftermath for children whose parents ‘hid it from them’. I reassure parents that it’s best to tell them soon and usually they are looking to me for the words to do that. [Interview 46, acute specialist, clinical nurse specialist].

From the parent data, parents seemed to have been navigating unchartered waters as mum or dad’s death approached in the final weeks and days of life. From the parents’ perspective, it was important for them to ‘live in the moment’ throughout the EOL period. However, when reality ‘hit’ that the ill-parent was actively dying, it often become a form of ‘crisis management’, with significant stress placed on the well-parent to tell the children that death was imminent. The parent data identified how the well-parent felt ill-equipped to initiate this conversation with the children, rapidly searching for guidance. HSCPs did not report this insight as to what was happening in the family when mum or dad’s death became imminent at the end.

I didn’t know what I was supposed to tell them. I ordered books for the kids from Amazon to prepare them thinking he would get time in the and they came on the Sunday morning and well he died that morning. [Interview 06, bereaved mother].

Bereaved parents often reported they had not thought out how ‘dying might look’ and what role their children would have at this time. Many bereaved parents described their ‘shock’ of how hasty death approached: in that death ‘caught up’ and they were not ‘expecting it’. Alongside this, it was reported in the HSCP and parent data that many parents had a lack of understanding surrounding the physiological aspects of death. In all data sets, there was a lack of preparations made for the children when mum or dad was actually dying. As a result, when the funeral director became involved in the immediate bereavement period, they noted how the bereaved parent struggles to share the devastating news with their children that mum or dad has died.

Jen [bereaved parent] didn’t know how to tell her wee girl [aged 12] that her daddy had died. She didn’t know if she could use the word ‘died’, so was saying things like ‘daddy’s a star now’. It was as if Jen was looking at me saying ‘what am I supposed to do here. [Interview 69, funeral director].

### Theme 2: coping throughout the unfolding end of life experience

The EOL experience was a changing landscape for families, with ongoing and different needs throughout the trajectory. The following three subthemes appeared helpful for parents as they coped and navigated the EOL experience: (1) striving for everyday ordinariness, (2) maximising social networks and (3) ‘hope’.

#### Sub-theme 1: striving for everyday ordinariness

From the parent and HSCP data, it appeared that maintaining some sense of ordinariness and family routines despite mum or dad’s declining health was helpful. This included the children going to school and attending their usual extracurricular groups, where possible parents continuing with work and other usual happenings in the family. It seemed mum or dad’s cancer was ‘normalised’ within the family, and as symptoms progressed for the ill-parent, the family continued to adapt and adjust with happenings as best as possible.

We just continued as things were. We didn’t specifically do things like going to Disneyland, as life was busy with A Levels and transfer tests. We weren’t purposely chasing time thinking we had to do all these things, but just enjoyed time together. [Interview 11, bereaved father].

The parent and HSCP data identified that healthcare teams occasionally had a pivotal role in helping ill-parents continue with ‘parenting’, when they were physically weak and becoming frailer. This included facilitating days to attend events such as a football match or school sports day. This allowed an ‘everyday activity to happen’ and were treasured, bringing joy to the parents and children. However, the findings from parents and HSCPs showed that these facilitations were rare and were more likely to happen when a parent was in a specialist palliative care unit.

The mum really wanted to get to her wee boy’s sport’s day. I think it was his first one, and we tried to see if we could get her out for it but it didn’t happen in the end. [HSCP 50, community generalist, district nurse].

#### Sub-theme 2: maximising social networks

From the parent data, it appeared that frequently parents required supportive input from extended family members such as grandparents, to help with the practical aspects of parenting. This was especially instrumental in the final weeks and days of the ill-parent’s life. At this particular juncture, there was often a ‘lack of available parenting’ from both parents, as the well-parents’ attention was primarily focused on the caring duties and spending time with the dying parent. From the parent data, it seemed parents were less connected with their children at this stage. HSCPs did not acknowledge this period of decreased ‘connection’ for well-parents with their children.

Alan [ill-parent] never got out of hospital then and that was for about three weeks. I needed to be with him at that time. I wasn’t focused on anything else. I was lucky to have my dad help out with the kids. If it wasn’t for him, I don’t know how I’d of managed. [Interview 04, bereaved mother].

#### Sub-theme 3: ‘hope’

Reported in the parent and HSCP data, it appeared ‘hope’ in treatment and that derived from spiritual faith facilitated coping for parents throughout the EOL experience. While parents and HSCPs reported that parents were hopeful that treatment may extend life, it was identified in the bereaved parent data that well-parents were more realistic that death was inevitable, from observing an overall decline in ill-parent’s health as the illness progressed.

He was going on this new treatment, and we both still hoped that this treatment would have worked, but in the back of my mind I was being a bit more realistic with myself. So, to basically tell the kids, we didn’t tell them and we certainly didn’t say that daddy was going to die. [Interview 10, bereaved mother].

### Theme 3: tension and complexities at end of life

From the parent and HSCP data, there appeared to be a tension between parents and also between parents and HSCPs in relation to how best to prepare their children for parental death. This aspect of care appeared complex for HSCPs to navigate, especially if one parent felt the children should be informed that mum or dad was eventually going to die and the other believed it was protecting the children from upset by not telling them. It also seemed from parents’ and HSCPs’ perspectives that family-centred conversations regarding the children were less prioritised if there was hope pinned on life-extending treatment, especially if embarking on novel immunotherapies. Alongside this, HSCPs reported a lack of confidence, skill and time to facilitate these emotive conversations with parents on how best to support their children, when one of them was dying from cancer. It was identified in the parent and HSCP data that it was often the well-parent navigating difficult conversations with the children, such as telling them mum or dad is going to die and when the parent was actually dying.

I would like to tell them [children] the truth of it because I don’t think I am being fair to them. It is a wee bit like the elephant in the room, but I am not just sure that Joan [well-parent] and myself are at the same place about telling them. [Interview 23, father at end of life].

To be honest I think I’ve avoided getting really into that. I’m afraid of telling them the wrong thing. I suppose if I felt a bit more comfortable about that, I might open that up a little bit more. But I don’t want to just start a conversation and then not have time to take it forward. How do I navigate that. [Interview 48, acute specialist; clinical nurse specialist].

### Theme 4: preparing for the future

Data from parents and funeral directors acknowledged the importance of making preparations for the future before the parent died, as a supportive measure for the family during the EOL period and moving forward after the death. This included outlining funeral wishes and sorting out finances, passwords on accounts and mortgages. Most bereaved parents stated that they had not made adequate preparations for everyday ‘life’ after the parent had died (practically and financially) but on reflection would have liked to have done so. From the parents’ perspective, it was too difficult for them to consider making detailed preparations for after they or their spouse had died.

I was coming into the home which was in chaos. Dad was in a ‘flap’ and not in the headspace to make preparations. It was going in one ear and out the other. What I think became more distressing for him was he didn’t know what his wife would have wanted. [Interview 71, funeral director].

For those couples who did have conversations about the fact that mum or dad’s death was inevitable, this gave children ‘permission’ to share their worries and concerns, like ‘what will we do if I need to get a costume for Halloween?’, ‘who’s going to fix things around the house now?’, or ‘are you going to be in a coffin’? These conversations could only happen if the poor prognosis was acknowledged by parents and integrated into ongoing conversations. HSCPs reported limited insight into the importance for parents to make preparations for the future before the ill-parent died.

From the parent and HSCP data, it appeared that some HSCPs were encouraging parents to engage in memory activities, such as writing letters for the future, to help aid the child’s connectedness to the parent for after they have died. While some parents did engage in these activities, in reality it was often too painful for parents to consider not being around and part of their children’s future. This insight was not considered by HSCPs.

As for memory boxes, we didn’t do it. To be honest we probably wouldn’t have wanted to do that. They have lots of wonderful memories of their mum that we didn’t feel the need to put them into a box. [Interview 09, bereaved father].

## Discussion

Findings highlighted how parents are central to preparing and supporting their children for the death of their mum or dad. Parents are the gatekeeper to providing information to their children surrounding their parent’s poor prognosis and updating them throughout the EOL experience. While children cope and adjust better when they are prepared for the death of a parents, findings highlight that parents need guidance on how best to manage and involve their children throughout the EOL experience.

Although preparing dependent children for the death of mum or dad is one of the greatest challenges that a parent can face, earlier preparations provide parents with the opportunity to comfort and protect their children before the death, which could help facilitate a better bereavement experience for the children [29, 30]. Actively walking together through the EOL experience when the ill-parent is capable of ‘parenting’ provides an opportunity for mum and dad to ‘parent’ a child through one of the most awful life changing times their children are going to experience. Parents should be encouraged by HSCPs that there is ‘a window of opportunity’ for them to utilise their parenting instincts, by including the children in the EOL experience. While the initial sharing of the news will be upsetting for the children, one or both parents can ‘parent’ and support their children through this experience. From the parents’ perspective, this may help control for crisis management as mum or dad’s death becomes imminent in the final weeks and days of life and facilitates important moments together at the end [31, 32].

Studies have suggested there can often be tension between parents’ and parents and HSCPs surrounding realistic and unrealistic expectations at EOL [33, 34]. From this study, one of the key factors that may have impacted parents’ expectations was a lack of clear prognostic communication from HSCPs. A similar finding has been reported in the literature [35]. It may be that telling a parent of dependent children that they were eventually going to die from cancer was too big for HSCPs to ‘take on’ and that a more ‘comfortable’, less personally emotively demanding position for the professional was engaging in physical care needs [20]. However, HSCPs did not acknowledge that hope centred on treatment would eventually place significant demands on the well-parent as death approached at the end. There is a need for HSCPs to balance hope of novel treatments in prolonging life and providing parents with clear and honest information surrounding a poor prognosis, to ensure they understand the severity of their condition and have realistic timescales. Clear prognostication may help promote advanced preparations for the future [36].

It may be suggested HSCPs do not want to get too emotionally close to parents who are dying and have dependent children, as to do so could lead to burnout for the professional [22]. Other explanations may include HSCPs were too ‘busy’ to take the time to provide this aspect of care [37] or a lack of supportive working environments to offload the emotional impact of having challenging EOL conversations with parents of dependent children [22]. While this is a complex aspect of care, there is a need to promote HSCPs’ awareness of the importance of family-centred care and the challenges faced by many parents as they prepare their children for the death of a parent. This can include training to increase HSCPs’ knowledge and confidence to enable them to meaningfully engage and support parents at EOL in relation to their dependent children. Appropriate supervision may promote HCSPs’ resilience to undertaking this aspect of care, reduce burnout and promote job satisfaction in cancer and EOL care [38, 39].

### Study strengths and limitations

Although the sample was selected from Northern Ireland, which has become increasingly secular and ideologically more diverse, it is a relatively culturally homogenous population [19]. While only three parents at EOL were included this study, triangulating the data identified how the data from parents at EOL mapped and mirrored findings from bereaved parents. This study addresses a gap in the literature in that findings are reflective mothers and fathers [2]. Children were not included in this study; however, this paper acknowledges the importance of involving them in ‘family life’ when a parent is at EOL from cancer [2] and the central role of parents to including them in this experience.

## Conclusion

There is a need for honest and clear communication from HSCPs to parents surrounding the reality of a parent’s poor prognosis, to enable a systematic approach of preparing the children for the death of their mum or dad. While parents often feel ill-equipped to prepare their children for the death of a parent from cancer and desire supportive guidance from healthcare teams, this is a challenging aspect of care for HSCPs to provide. Consequently, family-centred care is often inadequate in practice. There is a need for HSCPs to reassure parents that by involving the children early in the EOL experience, when the ill-parent is ‘well enough’ to parent, enables them to be actively involved in supporting their child through one, if not the greatest life changing event. This enables the sharing of sadness, providing love and support that only a parent can. Earlier preparations are likely to prevent ‘crisis management’ as death becomes imminent in the final weeks and days of life and promote better adjustment for the children in the future.

## Supplementary Information

Below is the link to the electronic supplementary material.

Supplementary file1 (DOCX 22 KB)

## Data Availability

The data that supports the findings of this study are available at the Ulster University repository and available on request from the third author. The data are not publicly available due to privacy and ethical restrictions. The study passed the ethical committee review [REC: 17/SW/01550.

## Code availability

N/A.
